# Incidence of Cardiovascular Events in Hypertensive Patients Based on the Quantity of Major Risk Factors According to the Isfahan Cohort Study

**DOI:** 10.1155/ijhy/3743691

**Published:** 2025-05-20

**Authors:** Masoumeh Sadeghi, Reza Shokrani Foroushani, Erfan Sabouri, Mohammad Talaei, Nizal Sarrafzadegan, Shahram Oveisgharan, Erfan Sheikhbahaei, Hamidreza Roohafza

**Affiliations:** ^1^Cardiac Rehabilitation Research Center, Cardiovascular Research Institute, Isfahan University of Medical Sciences, Isfahan, Iran; ^2^Interventional Cardiology Research Center, Cardiovascular Research Institute, Isfahan University of Medical Sciences, Isfahan, Iran; ^3^Isfahan Cardiovascular Research Center, Cardiovascular Research Institute, Isfahan University of Medical Sciences, Isfahan, Iran; ^4^Center for Primary Care and Public Health, Blizard Institute, Barts and the London School of Medicine, Queen Mary University of London, London, UK; ^5^Rush Alzheimer's Disease Research Center, Rush University Medical Center, Chicago, Illinois, USA

**Keywords:** cardiovascular diseases, heart disease risk factors, hypertension

## Abstract

**Introduction:** Hypertension is the most prominent established risk factor for adverse cardiovascular outcomes. The influence of hypertension in combination with other major cardiovascular disease risk factors (CVD-RFs) on mortality and cardiovascular events has not been fully comprehended yet due to their overlapping and interconnected nature. This study was conducted to evaluate the impact of CVD-RFs quantity on the occurrence of cardiovascular events, CVD-related mortality, and all-cause mortality rates in hypertensive patients.

**Design and Method:** In a secondary analysis of the Isfahan Cohort Study, demographic information, anthropometric measures, and laboratory results of participants were extracted. During the 15 years of follow-up, all-cause mortality, CVD-related mortality, and the occurrence of nonfatal cardiovascular events were assessed by separate panels of experts. Data analysis was performed using Cox proportional hazard models to estimate adjusted hazard ratios (HRs) among normotensive and hypertensive individuals in two subgroups of 3 CVD-RFs and≥ 3 CVD-RFs.

**Results:** Among 5432 eligible participants, hypertensive patients (*n* = 1509) had 1.3, 2, and 1.4 times higher HRs for all-cause mortality, CVD-related mortality, and nonfatal cardiovascular events, respectively. Compared to the normotensives, HRs for the mentioned outcomes were 1.2, 1.7, and 1.3 for hypertensive participants with < 3 CVD-RFs and 1.7, 3.4, and 2.3 for hypertensive participants with≥ 3 CVD-RFs. These rises were shown to be highly significant (*p* = 0.003, *p* = 0.001) for CVD-related mortality and nonfatal cardiovascular events in hypertensives with ≥ 3 CVD-RFs compared with hypertensives with < 3 CVD-RFs.

**Conclusions:** Hypertension alone or combined with other CVD-RFs increases the chance of all-cause mortality, CVD-related mortality, and nonfatal cardiovascular events. Rises in the quantity of other CVD-RFs (specifically to≥ 3) result in highly significant increases in fatal and nonfatal cardiovascular events. Therefore, to reduce mortality and cardiovascular events, hypertensive patients should be thoroughly evaluated for coexisting CVD-RFs, aiming to limit the synergistic effects of multiple CVD-RFs by properly managing modifiable RFs.

## 1. Introduction

Hypertension, with a global prevalence exceeding 1 billion individuals, is estimated to contribute to approximately 10 million deaths annually [1]. Among hypertension's associated complications, cardiovascular disease (CVD) is the most important, as it remains the leading cause of death in developed countries where its prevalence continues to rise [2]. Hypertensive patients are known to have a markedly higher lifetime risk of developing CVD compared to individuals with normal blood pressure (BP) (63.3% vs. 46.1%), as demonstrated in a cohort of over 1.25 million patients without baseline CVD [3]. Furthermore, large-scale studies like the NHANES III and the Framingham Heart Study have firmly established hypertension as the most prominent risk factor for adverse cardiovascular outcomes [4, 5]. Additional CVD risk factors identified by prior research, including the INTERHEART study, encompass aging, male sex, family history of CVD, smoking, dyslipidemia, diabetes, obesity, diet, physical activity, alcohol consumption, and psychosocial factors [6].

Extensive evidence indicates that a significant proportion of cardiovascular events occur in patients with multiple simultaneous CVD risk factors, raising the theory that the combination of risk factors with each other amplifies the likelihood of cardiovascular events [4, 7]. Importantly, many of these mentioned risk factors are modifiable through therapeutic lifestyle changes or medical treatments [8–10]. Therefore, early identification and proactive management of these risk factors in patients who are estimated to be at higher risk for cardiovascular events are essential for improving outcomes and reducing mortality rates [8–10].

Although numerous population-based prospective studies have explored the effects of hypertension on CVD mortality [10–13], only a limited number have managed to emphasize the effects of hypertension on CVD in conjunction with other risk factors [14, 15]. This limitation arises from the overlapping and interconnected nature of these risk factors in addition to pronounced ethnic disparities in CVD risk factors and their synergistic effects on mortality [14–16]. As the influence of the quantity of CVD risk factors on mortality rates is not yet fully comprehended, particularly, in Middle Eastern populations [15], this study was conducted to determine the impact of the quantity of known CVD risk factors on mortality and occurrence of cardiovascular events in hypertensive patients, utilizing data from the Isfahan Cohort Study (ICS), which encompasses urban and rural populations across three counties in central Iran.

## 2. Materials and Methods

### 2.1. Study Design

This study is a secondary analysis of the ongoing ICS, in which, after obtaining informed written consent, 6640 individuals over the age of 35 years at baseline were enrolled and followed biennially from 2001 onward to determine the individual and combined impacts of various risk factors on the incidence of cardiovascular events. The rationale, detailed methodology, and main findings of the ICS have been previously reported [17].

In accordance with the Declaration of Helsinki on human research ethics, records from all the consenting participants of ICS, who had completed baseline evaluations and at least three follow-up assessments, were reviewed and selected for the study. Records lacking sufficient data on the main mentioning variables were excluded. Ultimately, 5432 patients remained eligible for our study population through the 15 years of follow-up.

### 2.2. Data Collection

Data regarding the participants' demographics (i.e., age, sex, and educational level), lifestyle (i.e., physical activity level, smoking status, and quality of diet), family history (of hypertension or any cardiovascular events), anthropometric measurements (i.e., body mass index, waist circumference, hip circumference, waist/hip ratio, and waist/height ratio), and laboratory results (i.e., fasting blood sugar [FBS], blood sugar 2 h after consumption of 75 g glucose [BS 2hPP], serum C-reactive protein [CRP] level, and fasting blood lipid profile including triglycerides [TG], total cholesterol [TC], high-density lipoprotein cholesterol [HDL-C], and low-density lipoprotein cholesterol [LDL–C]) on the baseline of the study were extracted.

Participants were categorized as hypertensive or normotensive based on systolic and diastolic BP measurements taken using a mercury sphygmomanometer, according to the guidelines issued by the American Heart Association [18]. The reported BP for each visit from the participant was averaged from two BP measurements taken at least 30 min apart. Individuals were classified as hypertensive if their recorded BP was 140/90 mm Hg or higher at any of the baseline or follow-up visits or if they had been previously diagnosed with hypertension and were receiving antihypertensive treatment. For each participant, quality of diet was determined from a 48-item food frequency questionnaire and was reported as the global dietary index (GDI) [19]. Physical activity levels were obtained through the previously validated International Physical Activity Questionnaire (IPAQ) [20].

Our follow-up variables were nonfatal occurrence of cardiovascular events, CVD-related mortality, and all-cause mortality through the 15 years of follow-up. Living participants were interviewed biennially regarding the occurrence of cardiovascular events, including ischemic heart disease (fatal and nonfatal myocardial infarction and sudden cardiac death) or stroke (fatal or nonfatal), and were asked to provide relevant medical records. For deceased participants, trained nurses carried out verbal autopsies by interviewing surviving family members of the deceased patients using a defined standardized questionnaire to gather information on medical history, symptoms, and circumstances preceding out-of-hospital death. To verify reported events, two separate panels of four cardiologists and neurologists, who were blinded to the subjects' risk factor profiles, reviewed all relevant documents, including death certificates and verbal autopsies, to make a final decision. Diagnoses of acute myocardial infarction, unstable angina, or stroke were made following guidelines from the American Heart Association/American Stroke Association [21, 22]. A more detailed explanation of age grouping and data collection specifics of our study is accessible in another published article from the ICS [23].

### 2.3. Statistical Analysis

All the mentioned data were entered using Epi Info, Version 7 (Centers for Disease Control, Atlanta, GA) and analyzed by SPSS software Version 26 (SPSS Inc, Chicago, IL). The normality of the data was assessed using the Kolmogorov–Smirnov test. Continuous variables were compared using Student's *t* test, while discrete variables were analyzed using the chi-square test or Fisher exact test, as appropriate. *p* values below 0.001 were considered statistically highly significant.

Occurrences of nonfatal cardiovascular events, CVD-related mortality, and all-cause mortality rates were compared between the two groups of normotensive and hypertensive patients. Cox proportional hazard models were used to estimate hazard ratios (HRs) for future cardiovascular outcomes, with adjustments made for confounding factors, including age, sex, body mass index, educational level, diabetes mellitus, family history of CVD, smoking, and TC/HDL ratio.

Moreover, occurrences of nonfatal cardiovascular events, CVD, and all-cause mortality rates were analyzed separately among two subgroups of hypertensive patients with < 3 CVD risk factors and hypertensive patients with ≥ 3 CVD risk factors. These CVD risk factors were determined as aging (> 50), male sex, obesity (BMI ≥ 30 kg/m^2^), high TC/HDL (> 5:1), Type 2 diabetes mellitus (a serum FBS level equal or more than 126 mg/dL or a patient previously diagnosed), current smoking (at least one cigarette per day), and family history of CVD (report of CVD events in any of their first-degree relatives aged < 55 years for man and < 65 years for woman). Collinearity diagnostics using the variance inflation factor (VIF) revealed moderate correlations between the evaluated risk factors but no evidence of harmful multicollinearity (VIF > 5.0), confirming the reliability of the regression model's estimates (Supporting Table 1).

Additionally, the impact of each mentioned risk factor on cardiovascular events and mortality in hypertensive patients was analyzed, with adjustments made for age, sex, and educational level.

## 3. Results

**Figure 1:**
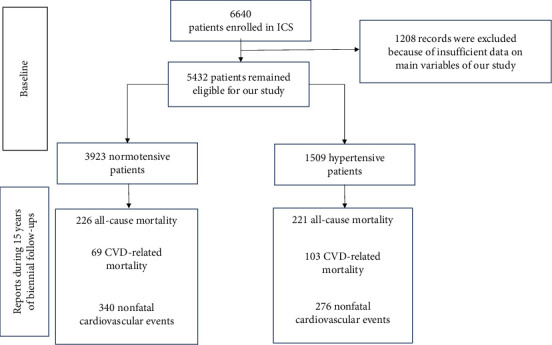
The diagram of the studied population and their outcomes.

In our study population of 5432 total eligible patients, the mean age was 50.6 ± 11.6 years old and 48% were male. The mean SBP and DBP measures of the population were 121.6 ± 20.9 and 78.3 ± 11.5, respectively, in which 27.8% of participants were considered hypertensive. During the 15 years of our evaluation, 616 patients experienced nonfatal cardiovascular events, and 447 patients were deceased, of which 172 deaths were determined to be related to cardiovascular events (Figure 1).

Our evaluation of CVD risk factors between hypertensive and normotensive patients revealed that female gender, older age, positive family history of high BP, heavier weight, higher anthropometric measures, more elevated levels of lipid profile, and blood sugar were significantly more likely to be involved in hypertensive patients (*p* value < 0.001). In contrast, smoking, higher diet quality index, higher levels of physical activity, and higher educational levels were more likely to be attributed to patients with normal BP (*p* value < 0.001). It was also shown that a positive family history of CVD and serum CRP levels did not have a significant difference between the mentioned groups (Table 1).

**Figure 2:**
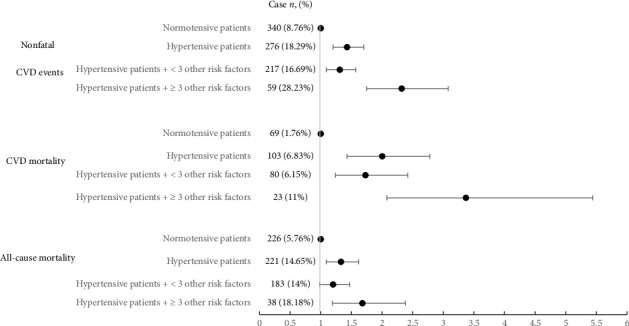
Hazard ratios (HRs) of incidence of all-cause mortality, CVD-related mortality, and nonfatal cardiovascular events in each group of normotensives, hypertensives in general, and hypertensives with < 3 or ≥ 3 cardiovascular disease risk factors based on Cox proportional hazard models.

Demonstrating the incidence and HR of cardiovascular events, CVD-related mortality, and all-cause mortality separately in two groups of normotensive and hypertensive patients (Table 2), the hypertensive group was subdivided into two groups of hypertensive patients with less than 3 CVD risk factors and hypertensive patients with at least 3 CVD risk factors. In order to achieve more precise results, HR was also reported in the form of adjusted for confounding variables (i.e., age, sex, educational level, diabetes, positive family history, BMI, smoking, and TC/HDL ratio). Patients with high BP had 43% more chance of experiencing nonfatal cardiovascular events, approximately two times more chance of dying from CVD, and 33% more chance of all-cause mortality in comparison to the normotensive population. Also, patients with less than 3 CVD risk factors other than high BP had 31% more chance of experiencing nonfatal cardiovascular events and 73% more chance of mortality caused by cardiovascular events in comparison to normotensive patients. Moreover, patients who had 3 or more CVD risk factors in addition to high BP had 132% more chance of experiencing nonfatal cardiovascular events, more than two times higher chance of mortality caused by cardiovascular events, and more than 1.5 times increased chance of all-cause mortality in comparison to normotensive people (Figure 2).

**Figure 3:**
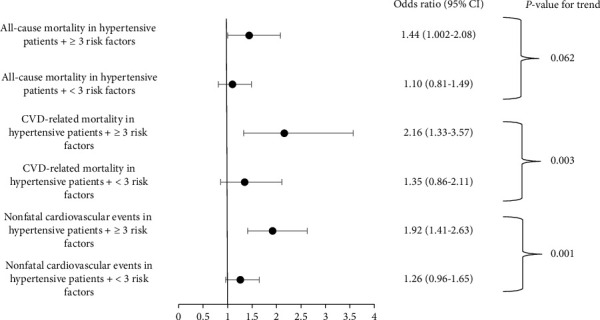
Comparison of all-cause mortality, CVD-related mortality, and nonfatal cardiovascular events between hypertensive patients with < 3 or ≥ 3 cardiovascular disease risk factors.

To evaluate the effect of the quantity of CVD risk factors on mortality rates and incidence of cardiovascular events more precisely, another comparison was made between patients with equal or more than 3 CVD risk factors other than hypertension and patients with less than 3 CVD risk factors. According to the results reported in Table 3, patients who had three or more CVD risk factors other than hypertension had significantly higher rates of mortality due to cardiovascular events and nonfatal cardiovascular events than those who had less than 3 CVD risk factors other than hypertension. The reported *p* value for the trend shows that as the number of CVD risk factors increases, the risk of mortality due to CVD and nonfatal cardiovascular events increases significantly (*p* = 0.003, *p* < 0.001) (Figure 3).

Finally, the effects of each CVD risk factor separately on the occurrence of cardiovascular events, CVD-related mortality, and all-cause mortality in hypertensive patients were evaluated and are summarized in Table 4. After controlling for confounding variables, it was shown that each year of aging increases the chance of incidence of nonfatal cardiovascular events, CVD-related mortality, and all-cause mortality rates by 3%, 9%, and 8%, respectively. Moreover, Type 2 diabetes mellitus in hypertensive patients increases the incidence of nonfatal cardiovascular events by 74%, CVD-related mortality by approximately three times, and all-cause mortality by approximately two times more than patients without this risk factor. In patients with hypertension, a high TC to HDL ratio only increased the rate of nonfatal cardiovascular events by 34%, and smoking only increased all-cause mortality risk by 61%. Male gender, obesity, and positive family history of CVD in hypertensive patients did not indicate a significant increase in nonfatal cardiovascular events, mortality due to CVD, and all-cause mortality.

## 4. Discussion

Our findings not only confirmed that rates of all-cause mortality, CVD-related mortality, and nonfatal cardiovascular events are higher in hypertensive patients but also showed that all these rates are increased significantly in the condition that three or more of the main CVD risk factors (i.e., age, male sex, obesity, high TC/HDL, diabetes mellitus, current smoking, and positive family history of CVD) coexist with hypertension. Moreover, by assessing the role of each mentioned risk factor solely, our study showed that aging and diabetes mellitus have the most significant effects on all the rates of all-cause mortality, CVD-related mortality, and nonfatal cardiovascular events, whereas smoking and high TC/HDL were only proven to have significant effects on all-cause mortality and nonfatal CVD events, respectively.

Hypertension, by inducing endothelial dysfunction and exacerbating atherosclerotic processes, is considered one of the most prevalent modifiable risk factors for premature CVD [24]. Global studies suggest that an estimated 54% of all strokes and 47% of all ischemic events due to heart disease are related to chronic elevation of BP [24]. The presence of other major CVD risk factors results in an increased rate of cardiovascular events, CVD-related mortality, and all-cause mortality, both directly and indirectly. Aging is considered one of the strongest determinants of CVD risk in many risk factor algorithms since it has many diverse effects on molecular, cellular, tissue, organ, and systemic levels of the body, contributing to an altered function of the cardiovascular system [25]. Showing the significant impact of age alone on CVD, in a cohort of more than 3.6 million individuals, it was demonstrated that with each additional decade of life, the risk of CVD gets approximately doubled [26]. Our study, confirming the results of other various cohorts, showed that aging, by itself, significantly increases cardiovascular events, CVD-related, and all-cause mortality [15, 27, 28].

The male sex has also been attributed to an increase in all major cardiovascular endpoints, probably due to variations in the Y chromosome as a risk factor for higher rates of CVD and CVD-related mortality [15, 29].

Several large cohort studies have demonstrated that conditions such as underlying genetic disorders leading to vasculopathies and coagulopathies are associated with a greater risk of developing CVD [30–32]. Therefore, even a self-reported positive family history in first-degree relatives can be of great value in the assessment of CVD risk [33].

Smoking is another major CVD risk factor that is considered to be associated with an increased risk of the occurrence of cardiovascular events, CVD-related mortality, COPD, cancer, and all-cause mortality [6, 34]. The impact of smoking on CVD is considered to be via inflammation, formation of free radicals, and carboxyhemoglobin, an increase in sympathetic activity (with increased myocardial oxygen demand and potential promotion of dysrhythmias), reduction in nitric oxide with endothelial dysfunction, oxidative stress, platelet reactivity, and coagulation [35].

Dyslipidemia plays a major role on the increased risk of cardiovascular events, CVD-related, and all-cause mortality due to an increase in atherogenic lipoproteins such as Apo B that may become entrapped within the subendothelial space and undergo oxidation and scavenging by arterial macrophages, resulting in endothelial dysfunction and formation of foam cells, fatty streaks, and atherosclerotic plaque. Progressive enlargement of the atherosclerotic plaque and its acute rupture may cause myocardial infarction or stroke [6, 14, 15, 27, 28, 36].

Obesity has also been proven to be a CVD risk factor associated with an increase in CVD and all-cause mortality by causing physiologic and metabolic changes, systemic inflammation, and endothelial dysfunction [37, 38]. The clustering of the most common metabolic diseases is due to an underlying same causality [39]. For example, the obesity epidemic is not only a known risk factor by itself but also a leading contributor to major CVD risk factors such as Type 2 diabetes mellitus, hypertension, and dyslipidemia that increase CVD and mortality rates separately [40].

Diabetes mellitus is another major risk factor responsible for an increased all-cause mortality rate, higher prevalence of nonfatal cardiovascular events, and CVD-related mortality, even after adjusting for advancing age, hypertension, smoking, and hypercholesterolemia [41, 42]. Hyperglycemia contributes to atherosclerosis via endothelial dysfunction, oxidative stress, heightened systemic inflammation, activation of receptors of advanced glycosylated end products, increased LDL oxidation, endothelial nitric oxide synthase (eNOS) dysfunction, and platelet hyperactivity [43].

The presence of coexisting CVD risk factors is common, and their combinations exert an additive effect on the likelihood of developing cardiovascular events and increasing CVD-related and all-cause mortality. In particular, the synergic effects of hypertension as a major CVD risk factor and other risk factors are proven to be of significant value in literature [11–13]. In 18,790 hypertensive patients from the Hypertension Optimal Treatment (HOT) Study, male gender, age > 65 years, diabetes mellitus, and smoking significantly increased the risk of major cardiovascular events, CVD-related, and all-cause mortality. Also, hypercholesterolemia was found to be a statistically significant risk factor for cardiovascular events and CVD-related mortality [15]. These synergic effects of risk factors were also proven in another cohort of 77,881 participants from India, in which it was shown that there is a higher rate of all-cause mortality among smokers or diabetic patients if hypertension is also present as a risk factor [44]. In another study conducted in 2020 following hypertensive participants on the incidence of fatal and nonfatal CVD events for 6.2 years, it was demonstrated that diabetes mellitus and serum cholesterol levels were significantly associated with an increased risk of cardiovascular events. Also, aging and diabetes mellitus were significantly associated with an increased risk of CVD-related mortality. Moreover, aging and serum cholesterol levels were found to be independent variables significantly associated with an increase in the all-cause mortality rate [28]. The study of Lee et al. on hypertensive patients from Singapore demonstrated that the presence of diabetes and smoking multiplied all-cause mortality rates by 1.7 and 2 times, respectively. Moreover, the presence of smoking, diabetes, and elevated TC/HDL-C significantly amplified CVD-related mortality rates by 1.9, 2.1, and 2.3 times, respectively, [14]. These results from the mentioned studies were mostly aligned with our findings on the subject of the collaborative effects of aging, diabetes mellitus, smoking, and dyslipidemia with hypertension on nonfatal cardiovascular events and CVD-related and all-cause mortality. However, we found the significance of gender, smoking, and hypercholesterolemia to some extent less for the events mentioned, which can be attributed to the difference in ethnicity, population size, years of patients' follow-up, and environmental factors.

The role of the quantity of CVD risk factors has already been evaluated in a couple of studies. In the Singapore Cardiovascular Cohort Study with 5830 participants followed for about 14 years, Lee et al. demonstrated that hypertensive participants with ≥ 2 CVD risk factors experienced higher rates of more than 2 times all-cause mortality and more than 4 times CVD-related mortality than those participants with < 2 CVD risk factors. They also reported a consistent trend of all-cause and CVD-related mortality risk elevation with the increase of the number of CVD risk factors in hypertensive participants [14]. In another study conducted in a population of 46,239 participants from China, the prevalence and odds ratio of self-reporting CVDs were compared among groups with various numbers of CVD risk factors (i.e., smoking, overweight/obesity, hypertension, dyslipidemia, or hyperglycemia). After adjustments for gender and age, the odds ratios of CVDs in those who had 1, 2, 3, or ≥ 4 risk factors were 2.36, 4.24, 4.88, and 7.22, respectively, compared with patients without any of the risk factors [10]. These results are in coordination with our findings, in which it is stated that as the quantity of known CVD risk factors increases, the risk of nonfatal cardiovascular events, CVD-related mortality, and all-cause mortality grows significantly.

### 4.1. Strengths and Limitations

Although our study's originality, relatively large population size, notable amounts of follow-up years, variety of assessed risk factors, and the strict overview of patient records for the certainty of cardiovascular events can be considered remarkable strengths, several limitations were unavoidable. These include possible measurement error of variables despite the recruitment of trained nurses and strict control of measurers, possible inaccuracy of self-reported information, remarkable loss-to-follow-up because of communicative and data keeping faults, lack of ethnic diversity, and external validity since the study was conducted in a restricted population and geographical area, and inability to consider the arising age-related physical issues due to unfeasibility of in-person follow-up interviews and laboratory test remeasurements.

## 5. Conclusion

High BP alone or in combination with other CVD risk factors increases the chances of all-cause mortality and fatal and nonfatal cardiovascular events. These rates also increase with the rise in the number of other CVD risk factors. To prevent cardiovascular complications and decrease mortality rates, more attention should be placed on hypertensive patients in early screening for hyperlipidemia and diabetes, smoking cessation, and effective BP management. Furthermore, additional research is needed to investigate the interactions and combinations of CVD risk factors to develop more precise and practical tools for CVD risk assessment.

## Figures and Tables

**Table 1:** Comparison of CVD risk factors, baseline demographics, and lifestyle data between hypertensive and normotensive participants.

	Normotensive (*n* = 3923)	Hypertensive (*n* = 1509)	*p* value
Demographic data			
Male	1995 (50.9%)	653 (43.3%)	< 0.001^△^
Age [Mean (SD)]	48.12 (10.5)	57.38 (11.75)	< 0.001∗
Educational Grade 1 (0–5 years)	1234 (31.4%)	744 (49.3%)	< 0.001^△^
Educational Grade 2 (6–12 years)	1403 (35.8%)	488 (32.3%)	< 0.001^△^
Educational Grade 3 (> 12 years)	1286 (32.8%)	277 (18.4%)	< 0.001^△^
Cardiac risk factors			
Family history of HTN	795 (20.3%)	413 (27.4%)	< 0.001^△^
Family history of early CVD	209 (5.3%)	82 (5.4%)	0.876^△^
TC [mean (SD)]	209.50 (50.59)	226.11 (54.57)	< 0.001^∗^
HDL [mean (SD)]	46.68 (10.31)	47.53 (10.46)	0.007^∗^
LDL [mean (SD)]	126.25 (42.54)	135.94 (44.98)	< 0.001^∗^
TG [mean (SD)]	182.85 (99.33)	213.18 (109.89)	< 0.001^∗^
TC/HDL [mean (SD)]	4.65 (1.36)	4.93 (1.48)	< 0.001^∗^
FBS [mean (SD)]	86.23 (29.85)	95.01 (38.88)	< 0.001^∗^
BS 2hpp [mean (SD)]	103.18 (43.80)	117.45 (55.38)	< 0.001^∗^
CRP [mean (SD)]	3.27 (1.43)	3.36 (1.70)	0.569^∗^
SBP [mean (SD)]	112.18 (11.91)	146.17 (19.31)	< 0.001^∗^
DBP [mean (SD)]	73.75 (7.82)	90.43 (10.94)	< 0.001^∗^
Anthropometric data			
BMI [mean (SD)]	26.24 (4.34)	27.86 (4.51)	< 0.001^∗^
Waist circumference [mean (SD)]	93.09 (12.004)	98.95 (11.96)	< 0.001^∗^
Hip circumference [mean (SD)]	101.06 (9.89)	103.71 (9.92)	< 0.001^∗^
Waist/hip [mean (SD)]	0.92 (0.07)	0.95 (0.07)	< 0.001^∗^
Waist/height [mean (SD)]	0.57 (0.08)	0.63 (0.08)	< 0.001^∗^
Lifestyle data			
Current smoker	733 (18.7%)	146 (9.7%)	< 0.001^△^
Diet quality index [mean (SD)]	0.99 (0.26)	0.91 (0.28)	< 0.001^∗^
Physical activity [mean (SD)]	915.72 (546.73)	759.60 (537/54)	< 0.001^∗^

*Note:* Physical activity = (MET-m/d).

Abbreviations: BMI = body mass index (kg/m^2^), BS 2hpp = blood sugar 2 h postprandial (mg/dL), CRP = C-reactive protein (mg/dL), DBP = diastolic blood pressure (mmHg), FBS = fasting blood sugar (mg/dL), HDL = high-density lipoprotein (mg/dL), LDL = low-density lipoprotein (mg/dL), SBP = systolic blood pressure (mmHg), TC = total cholesterol (mg/dL), TG = triglycerides (mg/dL).

^△^Fisher's exact test.

^∗^Independent Student's *t* test.

**Table 2:** Incidence of all-cause mortality, CVD-related mortality, and nonfatal cardiovascular events in each group of normotensives, hypertensives in general, and hypertensives with < 3 or ≥ 3 cardiovascular disease risk factors and the estimated hazard ratios△ (HRs).

		Case *n*, (%)	HR [95% CI] unadjusted	HR [95% CI] adjusted∗
All-cause mortality (*n* = 447 deaths)	Normotensive patients	226 (5.76)	1	1
	Hypertensive patients	221 (14.65)	2.61 [2.17–3.14]	1.33 [1.09–1.62]
	Hypertensive patients with < 3 other risk factors^•^	183 (14)	2.50 [2.06–3.04]	1.20 [0.98–1.47]
	Hypertensive patients with ≥ 3 other risk factors^•^	38 (18.18)	3.33 [2.36–4.69]	1.68 [1.19–2.38]

CVD-related mortality (*n* = 172 deaths)	Normotensive patients	69 (1.76)	1	1
	Hypertensive patients	103 (6.83)	4.01 [2.95–5.44]	2.003 [1.43–2.78]
	Hypertensive patients with < 3 other risk factors^•^	80 (6.15)	3.60 [2.61–4.97]	1.73 [1.24–2.42]
	Hypertensive patients with ≥ 3 other risk factors^•^	23 (11)	6.66 [4.15–10.68]	3.37 [2.08–5.44]

Nonfatal CVD events (*n* = 616)	Normotensive patients	340 (8.76)	1	1
	Hypertensive patients	276 (18.29)	2.04 [1.73–2.37]	1.43 [1.20–1.70]
	Hypertensive patients with < 3 other risk factors^•^	217 (16.69)	1.86 [1.57–2.21]	1.31 [1.09–1.57]
	Hypertensive patients with ≥ 3 other risk factors^•^	59 (28.23)	3.14 [2.37–4.15]	2.32 [1.75–3.08]

∗Adjusted for age, sex, BMI, educational level, diabetes mellitus, Family history of CVD, smoking, TC/HDL level.

^•^CVD risk factors are assumed as aging, male sex, obesity, High TC/HDL, Diabetes mellitus, positive family history of CVD, and Current smoking.

^△^Cox proportional hazard models were used to estimate hazard ratios (HRs) of hypertensive patients in two groups of with < 3 or ≥ 3 additional CVD risk factors for future cardiovascular events in comparison with normotensive patients.

**Table 3:** Comparison of all-cause mortality, CVD-related mortality, and nonfatal cardiovascular events between hypertensive patients with < 3 or ≥ 3 cardiovascular disease risk factors based on hazard ratios^△^ (HRs) and *p* value for trend†.

	Hypertensive patients with < 3 risk factors^•^	Hypertensive patients with ≥ 3 risk factors^•^	*p*-value for trend
All-cause mortality *n* (%)	183 (14%)	38 (18.18%)	0.062
HR [95% CI]	1.10 [0.81–1.49]	1.44 [1.002–2.08]	

CVD-related mortality *n* (%)	80 (6.15%)	23 (11%)	0.003
HR [95% CI]	1.35 [0.86–2.11]	2.16 [1.33–3.57]	

Non-fatal CV events *n* (%)	217 (16.69%)	59 (28.23%)	0.001
HR [95% CI]	1.26 [0.96–1.65]	1.92 [1.41–2.63]	

^•^CVD risk factors are assumed as aging, male sex, obesity, high TC/HDL, diabetes mellitus, positive family history of CVD, and current smoking.

^△^Cox proportional hazard models were used to estimate hazard ratios (HRs).

^†^ *p* values for trend were higher (*p* < 0.05) for CVD-related mortality and nonfatal cardiovascular events.

**Table 4:** Evaluation of the adjusted risk∗△ of all-cause mortality, CVD-related mortality, and non-fatal cardiovascular events in hypertensive patients in combination with other cardiovascular disease risk factors, separately.

		All-cause mortality n (%) HR [95% CI]	CVD-related mortality n (%) HR [95% CI]	Nonfatal CVD events n (%) HR [95% CI]
Hypertension with	Age	1.08 [1.07–1.10]	1.09 [1.06–1.11]	1.03 [1.04–1.1]
	Male sex	114 (17.46%)	56 (8.58%)	123 (18.84%)
		1.07 [0.81–1.41]	1.26 [0.84–1.90]	1.02 [0.79–1.32]
	Smoking	24 (16.44%)	10 (6.85%)	31 (21.23%)
		1.61 [1.03–2.52]	1.30 [0.66–2.58]	1.21 [0.81–1.82]
	Obesity	47 (10.47%)	23 (5.12%)	93 (20.7%)
		0.83 [0.59–1.1]	0.83 [0.51–1.3]	1.15 [0.8–1.4]
	High TC/HDL	90 (14.02%)	50 (7.79%)	143 (22.27%)
		0.8 [0.6–1.05]	1.09 [0.7–1.6]	1.34 [1.06–1.7]
	Diabetes	52 (26.64%)	33 (15.64%)	54 (25.59%)
		2.04 [1.4–2.7]	2.95 [1.9–4.5]	1.74 [1.2–2.3]
	Family history of CVD	8 (9.76%)	2 (2.44%)	18 (21.95%)
		0.75 [0.3–1.5]	0.39 [0.09–1.6]	1.06 [0.6–1.7]

^∗^Adjusted for age, educational level, and sex.

^△^Cox proportional hazard models were used to estimate hazard ratios (HRs).

## Data Availability

The raw data that were utilized in this study are available upon request from the corresponding author.

## Supporting Information

Supporting Table 1: Collinearity diagnoses based on variance inflation factor (VIF).
