# Supplementary material for: Incidence of Cardiovascular Events in Hypertensive Patients Based on the Quantity of Major Risk Factors According to the Isfahan Cohort Study
Source: Int J Hypertens. 2025 May 20;2025:3743691. doi: 10.1155/ijhy/3743691 (PMC12116197; doi:10.1155/ijhy/3743691)
Supplement: Supporting Information — Additional supporting information can be found online in the Supporting Information section. [file 3743691.f1.docx]

Supplementary Table-1: Collinearity diagnoses based on Variance Inflation Factor^✽^ (VIF)

| **Current smoking** | **Type 2 Diabetes Mellitus** | **Dyslipidemia** | **Obesity** | **Sex** | **Aging** | **Variables**^△^ |
| --- | --- | --- | --- | --- | --- | --- |
|  |  |  |  |  | 1.138 | **Sex** |
|  |  |  |  | 1.021 | 1.126 | **Obesity** |
|  |  |  | 2.820 | 1.423 | 2.875 | **Dyslipidemia** |
|  |  | 2.998 | 1.950 | 1.239 | 2.780 | **Type 2 Diabetes Mellitus** |
|  | 1.193 | 1.043 | 1.080 | 3.141 | 1.056 | **Current smoking** |
| 1.069 | 1.144 | 2.203 | 1.148 | 1.264 | 1.940 | **Family history of CVD** |

✽: Possible multicollinearity between the variables was quantified using the variance infiltration factor (VIF). A VIF > 5.0 was considered an indication of harmful multicollinearity in the regression model. VIF between 1 and 5 showed that variables were moderately correlated with each other.

△: The definitions of these cardiovascular disease risk factors were determined as:

Aging (>50 years old), Sex (Male), Obesity (BMI ≥ 30 kg/m^2^), Dyslipidemia (TC/HDL ratio > 5:1), Type 2 Diabetes Mellitus (Serum FBS level equal or more than 126 mg/dL or a previously diagnosed patient), Current smoking (One cigarette or more per day), and Family history of CVD (Report of CVD events in any of their first-degree relatives aged <55 years for man and < 65 years for woman).
